# Improving the Appropriateness of Referrals From Primary to Secondary Care Confounded by the COVID Era: Student Status and Quality of Referral Evaluation in Oxford City Team (SQUARE-OCT)

**DOI:** 10.1192/bjo.2022.93

**Published:** 2022-06-20

**Authors:** Wesley Quadros, Mohamed Ahmad, Wishwanath Patkee, Theodora Katsanouli, Katy Hyams, Nicola Watkins, Amani Krayem, Maja Bilip, Tarek Zghoul, Shah Tarfarosh, Leah Holm-Mercer, Sureyya Toparlak, Adam Tian, Khadija Masood, Digby Quested

**Affiliations:** 1 University of Oxford, Oxford, United Kingdom; 2 Oxford Health NHS Foundation Trust, Oxford, United Kingdom; 3 Oxford University Hospitals Foundation Trust, Oxford, United Kingdom

## Abstract

**Aims:**

The Oxford City and NE Oxon Adult Mental Health Team (AMHT) is an adult mental health team receiving referrals from GPs for most cases suspected to have a mental health illness requiring secondary mental health services’ input in Oxford city. In January 2020, the team was remodelled with care coordinators working in separate functions based on the duration AMHT support was required for, i.e. an assessment team and a treatment team, but with medics covering both functions of the team. This quality improvement project examines AMHT referrals over 2020/21, hypothesising a reduction in the proportion of inappropriate referrals following the remodelling compared to a 2018/19 pre-remodelling audit.

**Methods:**

The project covers a total of 2803 referrals the team has received from 13/01/2020 to 12/01/2021. The outcomes measured included the number of inappropriate referrals returned to the GP, referrals only requiring a single assessment, the proportion of these referrals as university students in Oxford, and the diagnostic groupings of the referrals in students vs non-students. These outcomes were measured pre- and during the COVID-19 pandemic over 2020/21.

**Results:**

A reduction in the total number of referrals to the team was noted over 2020/21 but this was compared to an 11 month audit in 2018/2019. During the study period, 19.5% (546/2803) of referrals were deemed inappropriate compared to 21% of referrals received in 2018/2019. Of 2803 referrals, 14.7% (97/658) were inappropriate pre-COVID-19 vs 20.9% (449/2145) during the pandemic. Of the total number of referrals, 32.9% were returned to the GP following a single assessment.

The top 3 diagnostic categories in ‘non-students’ were mood/affective disorders (33.7%), anxiety/stress related disorders (17.2%), and neurodevelopmental disorders (7.8% total - ADHD was 3%). A significant increase in ADHD referrals and mood disorders amongst students compared to non-students is notable with the top 3 diagnostic categories for students being mood/affective (24.7%), neurodevelopmental disorders (19.5% - ADHD 17.7%), and anxiety/stress related disorders (13.4%). Students constituted 26% of the total number of referrals.

It was notable that during the pandemic there was a higher proportion of inappropriate referrals.

**Conclusion:**

Our project demonstrates a reduction in the proportion of inappropriate referrals sent to the AMHT following remodelling as compared to 2018/19. Further work is necessary to elucidate the contributing factors and reduce inappropriate referrals even further. An innovation is planned to automate the logging of referral outcomes to expedite a re-audit.

